# Insight into the *bZIP* Gene Family in *Solanum tuberosum*: Genome and Transcriptome Analysis to Understand the Roles of Gene Diversification in Spatiotemporal Gene Expression and Function

**DOI:** 10.3390/ijms22010253

**Published:** 2020-12-29

**Authors:** Venura Herath, Jeanmarie Verchot

**Affiliations:** 1 Department of Plant Pathology and Microbiology, Texas A&M University, College Station, TX 77802, USA; venura.herath@tamu.edu; 2 Department of Agricultural Biology, Faculty of Agriculture, University of Peradeniya, Peradeniya 20400, Sri Lanka

**Keywords:** bZIP transcription factor family, *Solanum tuberosum*, drought stress response, heat stress response, virus stress response, gene regulatory networks, RNA sequence analysis, stress physiology and genetics, potato genome, potato transcriptome, genomic survey, tuber genome

## Abstract

The basic region-leucine zipper (bZIP) transcription factors (TFs) form homodimers and heterodimers via the coil–coil region. The bZIP dimerization network influences gene expression across plant development and in response to a range of environmental stresses. The recent release of the most comprehensive potato reference genome was used to identify 80 *StbZIP* genes and to characterize their gene structure, phylogenetic relationships, and gene expression profiles. The *StbZIP* genes have undergone 22 segmental and one tandem duplication events. Ka/Ks analysis suggested that most duplications experienced purifying selection. Amino acid sequence alignments and phylogenetic comparisons made with the Arabidopsis *bZIP* family were used to assign the *StbZIP* genes to functional groups based on the Arabidopsis orthologs. The patterns of introns and exons were conserved within the assigned functional groups which are supportive of the phylogeny and evidence of a common progenitor. Inspection of the leucine repeat heptads within the bZIP domains identified a pattern of attractive pairs favoring homodimerization, and repulsive pairs favoring heterodimerization. These patterns of attractive and repulsive heptads were similar within each functional group for Arabidopsis and *S. tuberosum* orthologs. High-throughput RNA-seq data indicated the most highly expressed and repressed genes that might play significant roles in tissue growth and development, abiotic stress response, and response to pathogens including *Potato virus X*. These data provide useful information for further functional analysis of the *StbZIP* gene family and their potential applications in crop improvement.

## 1. Introduction

Broad networks of transcription factors (TFs) exert basal control of gene expression, acting at the core promoter and engaging with RNA polymerase to initiate transcription. Regulatory TFs bind proximal and distal promoter regions to stimulate gene expression in a spatiotemporal and tissue-specific manner. They often recognize consensus sequences in promoters and can act on multiple genes. Such regulatory TFs are the master controllers of transcription networks and are fundamental for plant growth, development, and responses to environmental factors [1,2].

The basic leucine zipper (bZIP) domain TFs exist in all eukaryotes but remains one of the largest groups of TFs in plants [3]. The bZIP family is central to the regulation of developmental and physiological processes as well as abiotic and biotic stress responses [4]. The basic region of 16 amino acid residues contains a nuclear localization signal and an N-x7-R/K motif that binds DNA. The leucine zipper resides toward the C-terminus within an alpha-helical domain and drives protein dimerization [5]. The dimerization of two bZIP factors enables DNA binding [6,7].

Jakoby et al. (2002) were the first to report the Arabidopsis bZIP family contains 75 unique members that classify into ten phylogeny groups [8]. Droge-Laser et al. (2018) updated the classification and described 78 members of the bZIP-family in Arabidopsis and identified 13 groups [4]. The functional groups participate in gene regulation in response to abiotic stress, systemic acquired resistance to a broad spectrum of pathogens, energy metabolism, hypocotyl development, endoplasmic reticulum stress response, abscisic acid response, and virus infection. Plant bZIPs bind to an ACGT core sequence within an A-box (TACGTA), C-box (GACGTC), and G-box (CACGTG) [9].

The bZIP TF family is conserved across eukaryotes and has undergone intensive gene expansion in angiosperms [10]. Diploid angiosperms have undergone at least one duplication event and, polyploids have undergone more duplication events [11,12], making the number of bZIP family members in angiosperms larger than in Drosophila (27 bZIP members) or homo sapiens (53 bZIP members). The number of bZIP family member genes in Arabidopsis is 78, barley is 89, maize is 125, poplar is 99, rapeseed is 247, rice is 89, sorghum is 92, and soybean is 131 [4,10,13,14,15,16,17,18,19]. This expansion is attributed to the whole genome, segmental, and tandem duplications. Being sessile, plants have a robust need for expanded stress adaptation. While expanding the number of bZIP family members is one way to broader adaptation, another way to expand stress adaptation can be achieved through complex regulation of gene expression.

Potato, *Solanum tuberosum*, ranks in the top four most important crops in the world [20]. Potatoes are grown in all environments, latitudes, and hemispheres of the world. With climate change, there is a need to breed new potato varieties that can handle changing environments and warmer temperatures. There is also a need to understand the landscape of gene families that influence growth, development, and adaptive stress responses. The current available complete genome sequence and genome annotation [21] known as DM v6.1 allowed us to carry out a comprehensive identification and analysis of the *StbZIP* gene family and to conduct comparisons with the Arabidopsis *bZIP* gene family. In this study, we screened the most recent reference genome assembly and identified 80 *StbZIP* genes. We provide functional and regulatory classification groups based on the framework for classification of Arabidopsis bZIP family members. This study reports on the analysis of *StbZIP* gene expression profiles to understand the general involvement of the bZIP TF family in biological processes in *Solanum tuberosum*.

## 2. Results and Discussion

### 2.1. Identification and Phylogenetic Analysis of bZIP Family Members in S. tuberosum

We identified 67 *bZIP* genes in the *S. tuberosum* genome (EnsemblPlants SolTub_3.0 assembly) by restricting our query to the predicted domain model (IPR004827). We also identified thirteen additional *bZIP* genes in the SpudDB (DM1-3 R44 v6.1) which has the newest genome assembly (released September 2020) with higher coverage and resolution (Table 1 and Table S1). We performed reciprocal blast, phylogenetic comparisons, and sequence comparisons between the putative *StbZIP* family and the well-studied, 78 unique members of the *AtbZIP* family [4,8,10]. We designated the *S. tuberosum* genes as *StbZIP1* to *StbZIP80* according to the corresponding *AtbZIP* homolog or their locus ID. Table S1 is a compilation of the current locus IDs referenced in the 2020 updated DM v6.1 assembly and SolTub_3.0. We analyzed the amino acid (aa) length, molecular weight (MW), and protein isoelectric (PI) points of the StbZIPs. The StbZIP lengths vary from 91 aa to 876 aa, the MWs range from 10.11 to 96.59 kDa, and the PIs ranged from 4.56 to 10.15 (Table S1).

**Figure 1:**
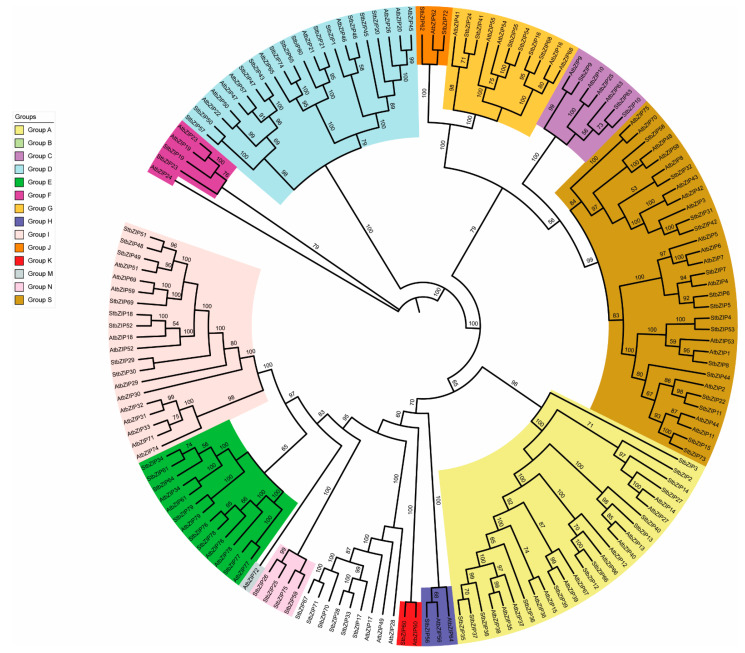
Phylogenetic tree constructed using PhyML method (v1.5) and Seaview (v4.7) contains the Arabidopsis and potato basic region-leucine zipper (bZIP) proteins. iTOL (v4) was used to visualize the output, and the diagrams were compiled, labeled, and color-coded using Adobe Photoshop CC (2017). Branch support was assessed with 1000 ultrafast bootstrap approximation and SH-aLRT branch test with 1000 replicates. The legend contains the functional groups assigned according to [8,10]. The bootstrap scores are provided at each node.

The available literature across angiosperms using neighbor-joining (NJ) and maximum likelihood (ML) phylogenetic analyses has revealed bootstrap support for thirteen groups of unique bZIP transcription factors [8,10]. According to this convention, we performed ML analysis to examine the congruences between the AtbZIP and StbZIP factors and provided the classification of the StbZIP-family within the prescribed 13 functional groups A through M, and S (Figure 1). We identified an additional functional group N that contains four StbZIP proteins. The phylogeny shows three deeply rooted branches. The first deep branch bifurcates with group F separating from all other groups. The second bifurcation separates group D, and the third branch expands to all remaining groups. The third branch also bifurcates. One branch extends to groups A, B, E, H, I, K, and N. The other branch extends to groups C, G, J, and S. Each group shows a pattern of AtbZIPs and StbZIPs sharing a common shallow node suggesting these gene pairs are likely orthologs. Other internal nodes seem to represent two or more paralogs suggestive of gene duplication events (Figure 1).

Table 1 shows the gene names and identifiers for the *AtbZIPs* and *StbZIPs* used in this study. The biological functions of the AtbZIP protein groups have been extensively studied [4] and used here as the framework for our categorization and discussion of StbZIPs. There are thirteen AtbZIP and thirteen StbZIP members of group A [22]. The Arabidopsis group A includes AtbZIP14/FD, and the paralog AtbZIP27/FD is mainly expressed in the shoot apical meristem and is involved in flowering time. The abscisic acid (ABA)- responsive element (ABRE) binding protein or ABRE binding factor (ABF), and the ABA insensitive/*Dc3* promoter-binding factors (ABI/DPBF) (Table 1) are essential for stress responses under conditions of dehydration, salinity, or osmotic stress. Mutations in these genes alter plant resiliency to drought, salinity, and osmotic stress. The ABI/DPBF factors regulate seed germination [4,8,23,24]. The *S. tuberosum* orthologues, which were assigned synonyms in a recent study [22], include StbZIP12, StbZIP35, StbZIP36, StbZIP37, StbZIP38, StbZIP39, and StbZIP66. Group B and group K are closely related and include AtbZIP17, AtbZIP28, AtbZIP49, and AtbZIP60 and have in common the presence of a transmembrane domain [25]. The Arabidopsis TFs form homodimers and heteromers and regulate endoplasmic reticulum (ER) stress. Their transmembrane domains facilitate interactions with other cellular proteins such as AtBAG-7 and BiP [26,27,28]. The AtbZIP28 also interacts with NF-Y subunits to form a transcription complex suggesting some promiscuity among these group members [29,30,31]. *S. tuberosum* has a single ortholog of the AtbZIP60 in group K and six group B factors StbZIP17, StbZIP28, StbZIP33, StbZIP67, StbZIP70, and StbZIP71 (Figure 1). Expansion of *StbZIP* genes in group B likely occurred by gene duplication [11,12].

The Arabidopsis literature [4,31] describes group C and S as sister clades that cooperate for some biological functions (Figure 1). For Arabidopsis, group S subdivides into subgroups S1, S2, and S3 [31]. The C/S1 network regulates genes that respond to energy starvation, express primarily in sink tissues, are involved in seed and pollen development, and control the expression of seed storage proteins (SSPs) [32,33,34]. For Arabidopsis, the C and S1 factors do not generally form homodimers, are not promiscuous and, have specificity in their interactions with each other, which is why this is known as the C/S1 gene regulatory network [35].

In the phylogenetic analysis of StbZIPs reported in Figure 1, the group C StbZIP9, StbZIP10, and StbZIP63 share a node with two AtbZIP factors; AtbZIP9 and AtbZIP63. There does not appear to be a direct orthologue in *S. tuberosum* for AtbZIP25. The Arabidopsis subgroup S1 consists of AtbZIP1, AtbZIP2, AtbZIP11, AtbZIP44, and AtbZIP53, and *S. tuberosum* has eight S1 members identified as StbZIP4, StbZIP8, StbZIP11, StbZIP15, StbZIP22, StbZIP44, StbZIP53, and StbZIP73. In total, group S has 17 Arabidopsis bZIPs and 15 StbZIPs, which suggests that some genes were lost in potatoes during angiosperm evolution. This loss may relate to different genetic programming in potatoes needed to produce tubers with buds for asexual reproduction. Group D has twelve StbZIP members and includes the Arabidopsis TGACG-binding (TGA) factors, which contribute to cellular defenses [36]. Groups E, F, and G have seven, two, and six StbZIP members, respectively. StbZIP61 is in group E and functions in salicylic acid signaling and defense against *Phytophthora infestans* [37]. Group H has factors involved in light regulation and anthocyanin accumulation and has one StbZIP member. Groups I, J, M, and N contain eight, one, zero, and four StbZIP members, respectively. Group G and H consist of Arabidopsis members that primarily form homodimers for their group [38].

### 2.2. Chromosomal Distribution of StbZIPs and Analysis of Gene Duplication Events

**Figure 2:**
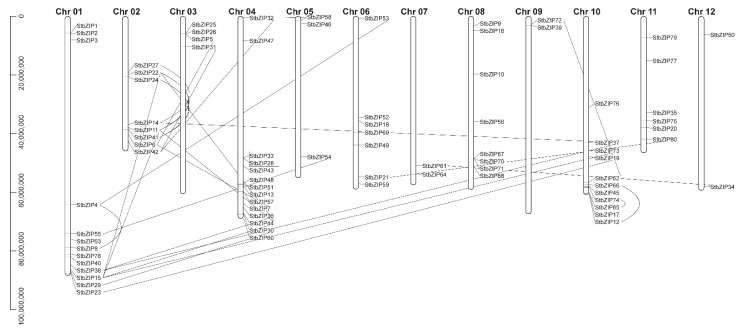
Distribution and segmental duplication of *StbZIP* gene family members in *S. tuberosum* chromosomes. The location of *StbZIP* genes on twelve chromosomes. The scale indicates the genome size of S. tuberosum (Mb). Bold lines connect paralogs, and segmental duplication was identified using MCScanX methods. The box identifies two genes representing a tandem duplication event.

We mapped these 80 *StbZIP* genes onto the 12 chromosomes (Figure 2) [22]. Thirteen *StbZIPs* map to Chr 1, eight *StbZIPs* map to Chr 2, four *StbZIPs* map to Chr 3, fourteen *StbZIPs* map to Chr 4, three *StbZIPs* map to Chr 5, seven *StbZIPs* map to Chr 6, two *StbZIPs* map to Chr 7 and Chr 12, eight *StbZIPs* map to Chr 8, two *StbZIPs* map to Chr 9, eleven *StbZIPs* map to Chr 10, and six *StbZIPs* map to Chr 11. We did not see an apparent pattern of members belonging to specific functional groups clustering on chromosomes.

Gene duplication analysis was carried out using MCScanX. We identified 22 *StbZIP* paralogs (27.5%) that arose by segmental duplication. We calculated the nonsynonymous mutation rate (K_A_)_,_ synonymous mutation rate (K_S_), and K_A_/K_S_ values to study pairs of *StbZIP* paralogs and to understand the selection pressures affecting sequence divergence. A K_A_/K_S_ >1.0 indicates positive selection, a K_A_/K_S_ = 1.0 indicates neutral selection, and a K_A_/K_S_ < 1.0 indicates negative or purifying selection. Among the *StbZIP* pairs tested, the K_A_/K_S_ values (<1) were between 0.14 and 0.48, suggesting purifying selection. Only the *StbZIP28* and *StbZIP33* paralogs appear to be the result of tandem duplication (Table 2).

### 2.3. Gene Structure Analysis of StbZIP Genes

**Figure 3:**
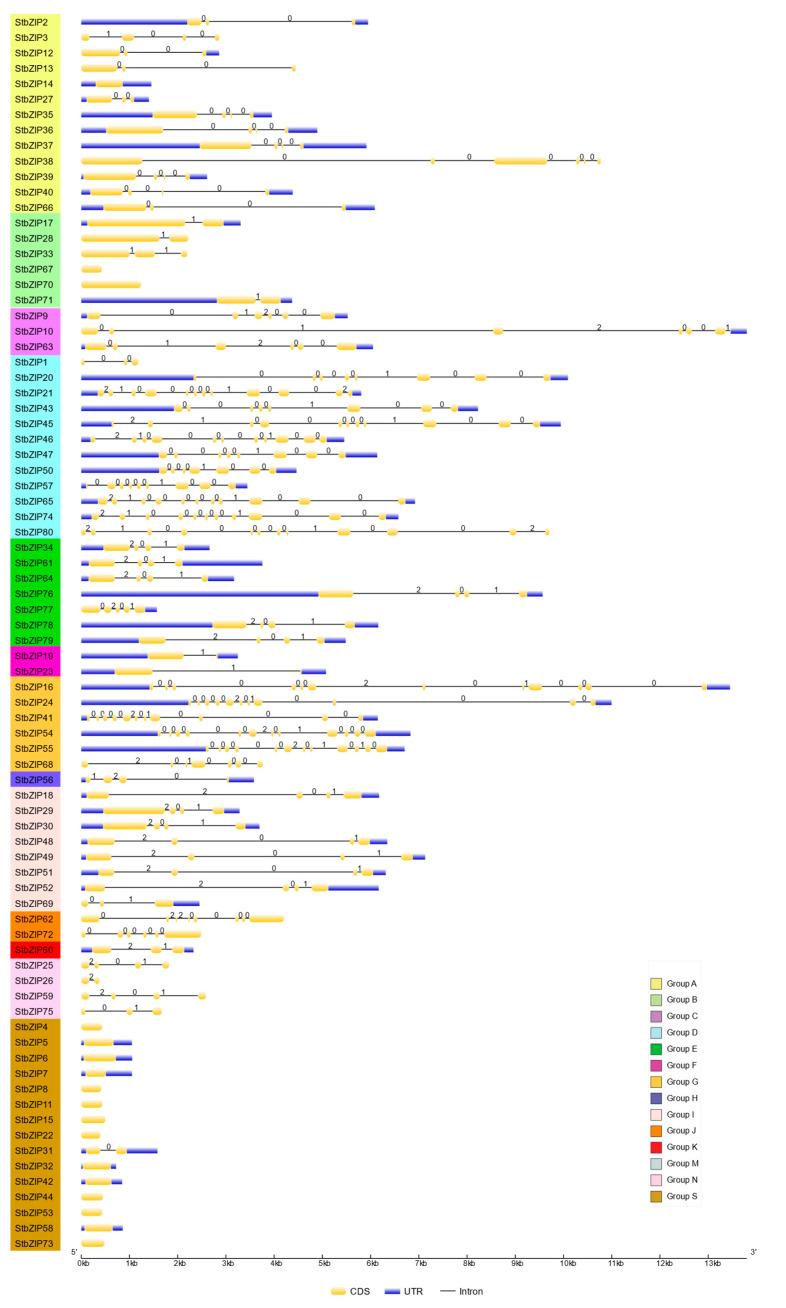
Exon/intron organization of *StbZIP* genes depicted for each group. The legend in Figure 2 explains the color scheme highlighting gene names. Boxes represent exons, and lines represent introns. The numbers “0”, “1”, and “2” identify the splicing phases.

The position of introns within a codon phase 0,1, or 2, were mapped for each *StbZIP* gene (Figure 3) because the intron positions and the frequency of intron phase combinations in related genes can be evidence of a common progenitor. A non-random pattern of introns indicates that they were acquired from a progenitor and stabilized through evolution [39]. Random phase distribution of introns suggests exon shuffling through evolution, which is typically evidence of new functional elements of protein gene products.

The representative coding sequences (CDS) of the 80 *StbZIP*s vary from 274 bp to 2631 bases and were used to map the boundaries of genes for structural analysis. The group A genes range from zero to five symmetric exons (0,0), except *StbZIP3,* which has one asymmetric (1,0) exon. Group B has zero, one, or two phase 1 introns. Group C has three genes with the same pattern of three asymmetric exons (0,1), (1,2), and (2,0) and a 3’ symmetric exon (0,0). *StbZIP10* has an added phase 1 intron at the 3’ end. Group D has two intron phase patterns. First is *StbZIP1, StbZIP20, StbZIP43, StbZIP47, StbZIP50, StbZIP57* whose 5’ ends have two symmetric exons (0,0) and the 3’ ends have one symmetric exon (0,0) and a central asymmetric exon (0,1). The second pattern is represented by *StbZIP14, StbZIP30,* and *StbZIP44*, which have added asymmetric exons at the 5’ end (2,0). The *StbZIP80* is an asymmetric exon at the 3’ end (0,2). Group E has two asymmetric exons (2,0) and (0,1), except for *StbZIP77*, which has an additional 5’ asymmetric exon (0,2). Group F has one phase 1 intron. Group G genes have four central asymmetric exons (0,2), (2,0), (0,1), (1,0) and adjoining one or two 3’ symmetric (0,0) exons. The 5’ has between two and four symmetric (0,0) exons. Group H has two asymmetric exons (1,2) and (2,0). Group I has two asymmetric exons (2,0) and (0,1) except for *StbZIP69*, which has (0,1). Group J has two genes with different 5’ exon patterns and three symmetric 3’ exons (0,0). Group K has a simple asymmetric (2,1) exon pattern. Group N has asymmetric (2,0) and (0,1) exons. The Arabidopsis and potato group S members are unique intronless genes [35,40], except for *StbZIP31,* which has a single phase 0 intron. Overall, the common intron patterns and phases provide further support for segmental duplication as the major mechanism for gene expansion (Figure 3).

### 2.4. Analysis of the Amino Acid Composition Among Leucine-Rich Repeats belonging to S. tuberosum bZIP TFs

**Figure 4:**
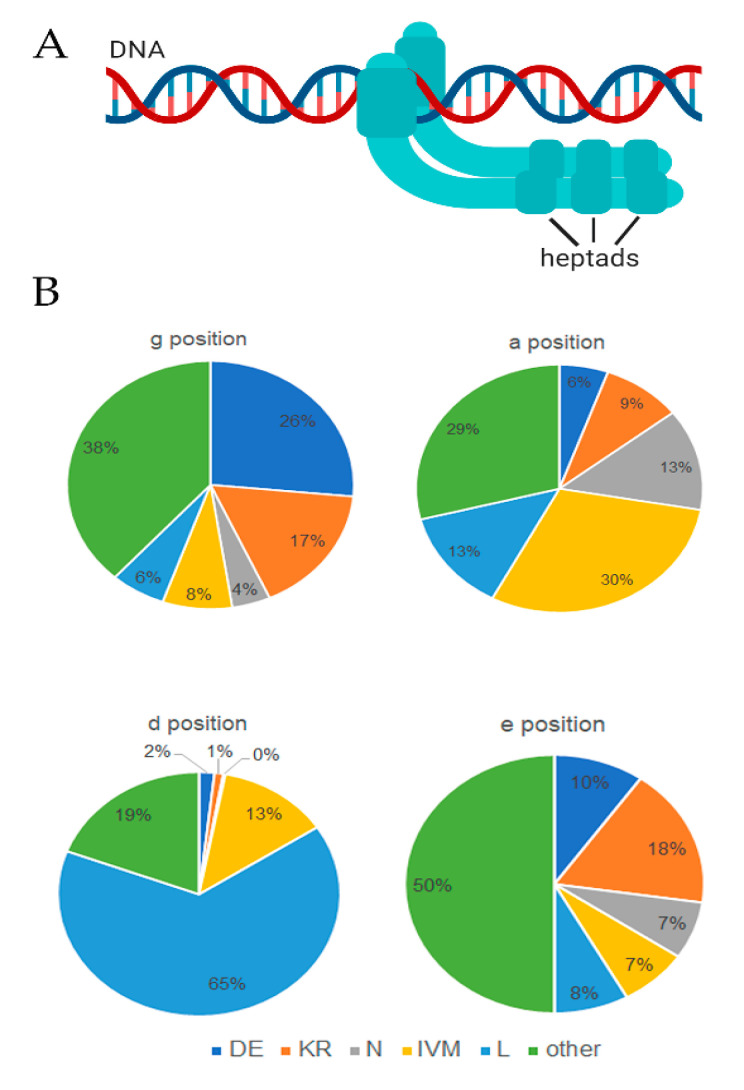
Analysis of amino acid composition for heptads. (**A**) Depiction of bZIP transcription factor binding DNA and dimeric heptads. (**B**) Pie chart presenting the frequency (%) of amino acids in the g, a, d, e position of heptads from the 80 StbZIP proteins.

To further validate the genes that we identified in this study encoding bZIP factors, we analyzed the amino acid composition of the basic and leucine-rich repeat domains which define the bZIP family. The leucine zipper governs dimerization and has a structural repeat of heptads. It is standard to designate the amino acid positions using letters *a*, *b*, *c*, *d*, *e*, *f*, and *g* [6,7]. For Arabidopsis, the bZIP- TFs vary between three heptads and ten or more heptads (Figure 4A) [7,33]. A region of basic amino acids (N X (7) R/K) defines the N-terminal boundary of the leucine zipper. The C-terminal limit can be identified by the presence of proline or other amino acids that disrupt the alpha-helical structure and follow a heptad with the leucine in the *d* position [6,7]. Identifying the C-terminal disruptor can be difficult based on the amino acid sequence alone. Prior structural studies identified important amino acid features that are essential to forming a productive dimerization interface. For example, the *g* and *e* positions typically contain charged residues D, E, K, or R, which provide attractive or repulsive pairing in each heptad. Leucine typically occupies the *d* position, although other combinations of aliphatic residues can occupy the *a* and *d* positions to create the dimerization interface needed to stabilize the leucine zipper [6,33,41].

We assessed the amino acid content for the bZIP factors of *S. tuberosum* at the *a*, *d*, *e*, and *g* positions within the heptads L0 through L4 (Figure 4B). Sixty-five percent of heptads contained L in the *d* position, consistent with reports for humans and Arabidopsis [6,7]. Considering the *g* ⬄ *e’* pairs, the presence of D, E, K, and R, which most often drive attractive and repulsive interactions were 43% for the g position and 28% for the e position. Finally, the *a* ⬄ *a*’ pairs also provide an energetic contribution to leucine zipper stability. Homotypic valine, isoleucine, or arginine interactions are favorable, and charged amino acids can be destabilizing. In the ‘*a*’ position, N⬄V pairs favor heterodimerization, whereas N⬄I pairs are destabilizing. For potato, N, I, V, M occupy the ‘*a*’ position in 30% of heptads, and charged residues (K, R) are infrequent (Figure 4B). These data are within the realm of expectations for bZIP-family members reported for humans and Arabidopsis [6,7].

We aligned the amino acid sequences of the Arabidopsis and *S. tuberosum* bZIP factors. Within the bZIP domains, heptads were labeled using the standard designations of *a, b, c, d, e, f,* and *g*, counting from the first leucine in the d position of the L0 heptad (Table S2) [4,6,13,22]. The L1 and L2 heptads consist of attractive pairs favoring homodimerization, repulsive pairs favoring heterodimerization, or a combination of attractive and repulsive heptads. It is interesting to note that the patterns of attractive and repulsive heptads were similar in each group for Arabidopsis and *S. tuberosum* orthologs. Furthermore, the N residues in the ‘*a’* position of L2 heptads were often conserved between Arabidopsis and *S. tuberosum* bZIPs in the same group. These observations suggest that the experimental data documenting interactions among Arabidopsis bZIP proteins may be useful to predict the interactions among *S. tuberosum* bZIP proteins. There was a broad pattern of early heptads that were similarly attractive or repulsive across each family, although this pattern was not strictly maintained throughout adjoining heptads within each family (Table S2). Importantly the AREB/ABF/ABI5 transcription factors in group A have four characteristic phosphorylation sites (R-X-X-S/T) across the protein, including one known as the C4 site after the leucine-rich domain [8,22]. Also notable in the alignment, is the StbZIP14 in group A, StbZIP67 in group B, StbZIP1 in group D, and StbZIP26 in group N showed only two or three complete heptads and leucines were lacking in the heptads defining the leucine zipper. For example, the basic regions of StbZIP14 were highly conserved with group A members, but the three heptads did not have leucine in the *d* position, and the g⇔e’ pairs were not well conserved with other members of the family.

The StbZIP67 in group B has conserved basic region and L0 heptad but the L1 and L2 heptads lacked leucine in the *d* position. The StbZIP1 in group D lacked L in the d position of L2 and L3. The StbZIP26 in group N did not have leucine in the *d* position of L2 heptad (Table S2). Possibly, the sequences recovered by database mining produced candidates that show a high degree of similarity but require further refinement built on more extensive functional analysis in future studies. We expect that further refinement of the StbZIP family will be necessary considering that several studies surveying the Arabidopsis genome reported 74, 75, and 78 bZIP-family members [4,8,16]. The most recent report of 78 *AtbZIP*-family members derives from an enormous amount of functional data used to refine the list of family members [4].

AtbZIPs belonging to group C and subgroup S1 have eight hydrophobic repeats, higher than other groups [7,33]. These AtbZIPs form specific heteromers that cooperate in regulating seed maturation genes and nutrient allocation. Notably, the heptad patterns are conserved among group C and subgroup S1 AtbZIP and StbZIP factors (Table S2).

### 2.5. Analysis of Conserved Protein Motifs

**Figure 5:**
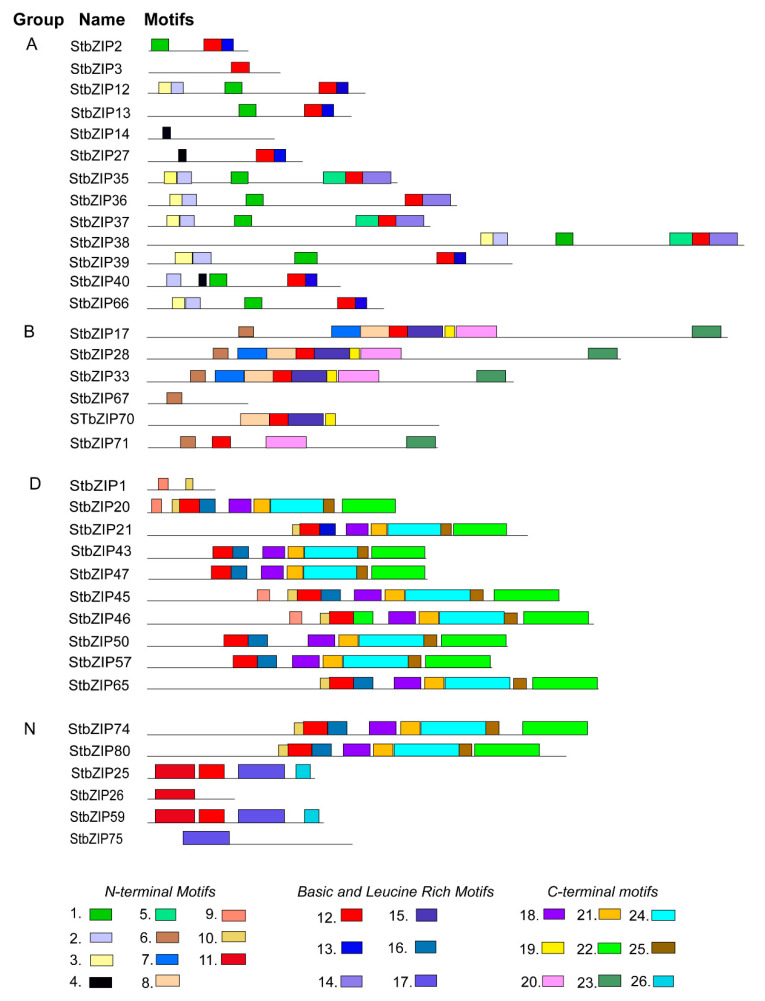
Conserved motifs identified using MEME software suite. All factors, except six, were predicted to have one bZIP domain (*p* < 0.001). The conserved basic N-X (7)-R/K motif is colored red, and adjoining leucine-rich motifs are shaded blue. Figure S1 provides the details for all motifs in this figure.

Grouping of bZIP family members is based on homology of the basic region and other conserved motifs. The heptad analysis identified the StbZIP14 in group A, StbZIP67 in group B, StbZIP1 in group D, and StbZIP26 were lacking heptads defining the leucine zipper. Therefore, we conducted a motif search using MEME Suite software to identify fixed pattern motifs that are present in proteins within these four functional groups (Figure 5). We identified the bZIP domains and labeled the conserved motifs lying toward the N- or C-terminus (Figure 5 and Figure S1). Notably, all groups have the same fixed bZIP segment consisting of a basic region (N-X(7)-R/K) and the L0 heptad, followed by longer leucine-rich motifs that were specific to each functional group. Group A members segregate into two clusters of conserved bZIP motifs. The first cluster is StbZIP2, StbZIP12, StbZIP13, StbZIP27, StbZIP40, and StbZIP66. The second cluster consists of StbZIP35, StbZIP36, StbZIP37, StbZIP38, and StbZIP39. These two clusters also occur in the functional group A of the phylogeny. We propose that the conserved motifs outside the bZIP domain account for the functional specificity of each major group (Figure 5).

Each group had at least one member that lacks a fixed basic and leucine-rich motif representative, although containing other conserved motifs. Specifically, the StBZIP14 of group A, StbZIP67 of group B, StbZIP1 in group D, and StbZIP26 of group N lacked the fixed basic and leucine-rich pattern motif. In addition, StbZIP3 in group A and StbZIP71 in group B have the conserved basic region but lack the leucine-rich sequences. Importantly, these genes were identified by InterPro as bZIP domain-containing proteins. Their phylogeny and gene structure shows significant conservation with other bZIP family members despite the MEME analysis suggesting that the leucine-rich sequences are not synonymous with these fixed motifs of related factors. Considering the results of motif analysis, six candidate StbZIP TFs may not have the same dimerization properties of other bZIP proteins.

### 2.6. StbZIP Promoters Enriched with Developmental, Hormone-Response, and Stress-Related TF Binding Sites

**Figure 6:**
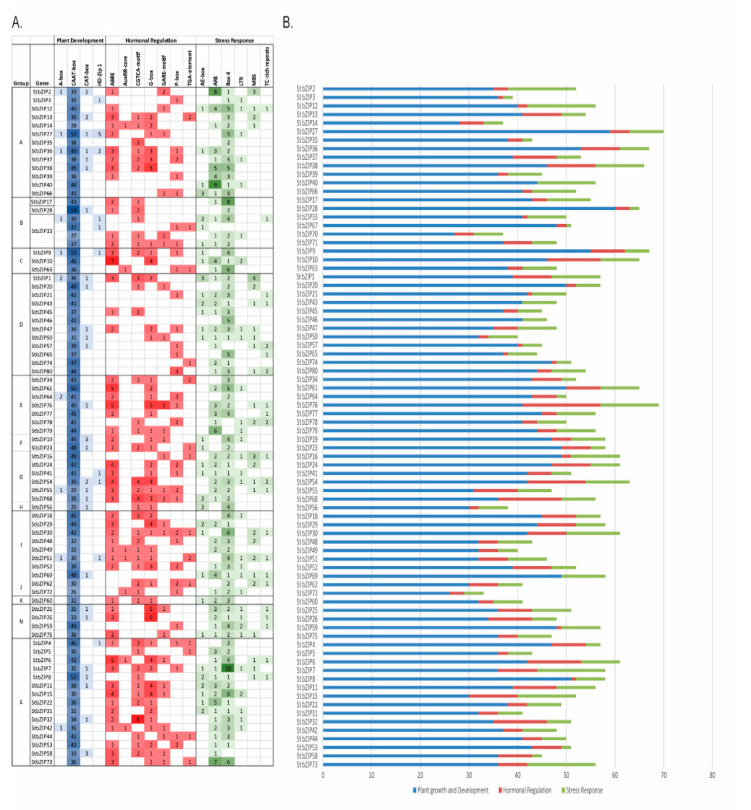
Distribution and frequency of cis-regulatory elements (CREs) on *StbZIP* promoters. Promoter sequences representing 2000 bp from the transcription start site of StbZIP genes were extracted from the DM1-3 516 R44 v 6.1 assembly at Spud DB as described in Materials and Methods Section 3.4. (**A**) The grid provides the number of sites that contain the CREs involved in the development, hormone signaling, and stress responses. Shades of green, red, and blue point to CREs that are highly represented (dark-colored), moderately represented (medium colored), once or twice represented (light color), or not present (white color). (**B*)*** Bar graph demonstrates the distribution frequency of CREs involved in the development, hormone signaling, and stress responses across the 2000 bp sequences analyzed for each *StbZIP*.

To identify the basis of differential gene expression, we selected 2000 bp upstream of the predicted transcription start site for the *StbZIP* promoters and derived the predicted cis-regulatory elements (CREs). We identified 18 transcription factor binding sites. The CAAT-box is a ubiquitous core element of eukaryotic promoters and is abundant in the *StbZIP* promoters. The number of CAAT-box sequences ranges from 25 to less than 60 among all *StbZIP* promoters (Figure 6A,B). Other elements such as the A-box, CAT-box, or HD-ZIP1 core elements occur in 10 and 27 promoters. The CAT-box and HD-ZIP elements are meristems and leaf regulatory elements. The abscisic acid-responsive elements (ABRE) are consistently represented in the majority of bZIP promoters, while only seven promoters have auxin-responsive elements (AuxRR-core). Most promoters contain between one and four copies of the MeJA-responsive and light response elements CGTCA-, G-box, and/or giberillic acid responsive element (GARE)- motifs (Figure 6A). Fourteen promoters have one or two TGA-elements (Figure 6A). There appear to be more stress-responsive elements than hormone regulating elements (Figure 6A,B). There are between 1 and 10 copies of the AE-box, ARE, and/or Box 4 elements across all StbZIPs. More than half the genes in each functional group have between one and four copies of the low temperature responsive element (LTR), MYB binding sequence (MBS), and TC-rich elements.

### 2.7. Differential Gene Expression in Plant Tissues 

**Figure 7:**
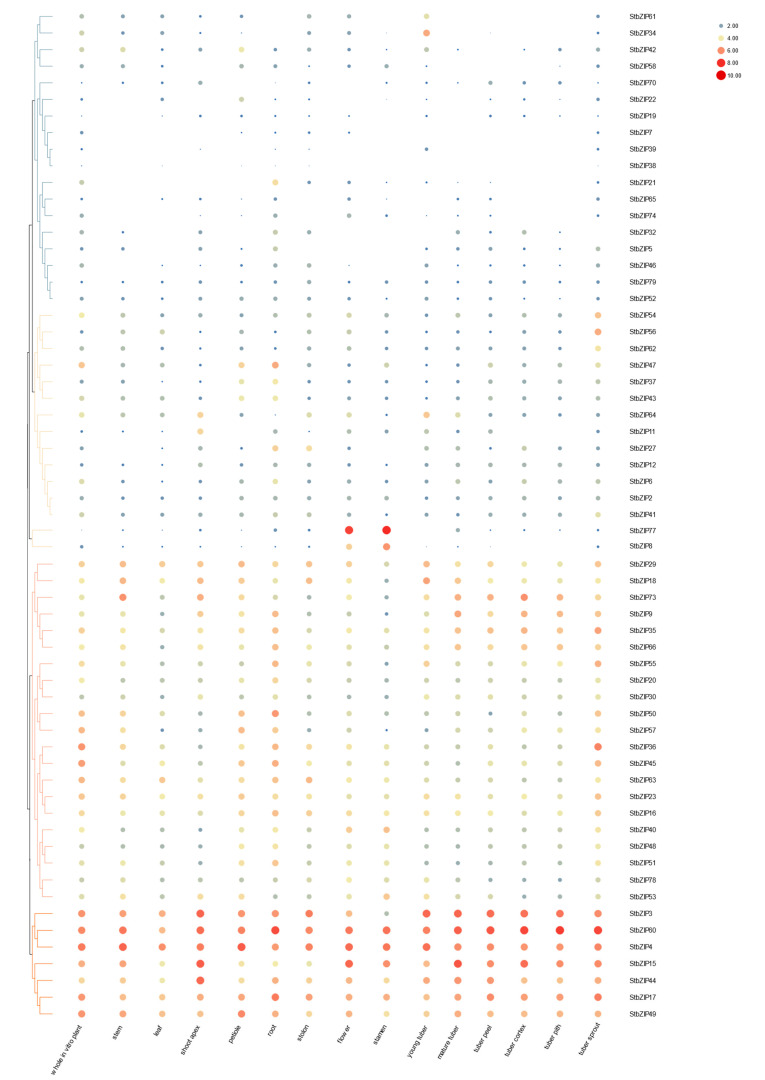
Heat map representation and hierarchical clustering of *StbZIP* transcript levels across different tissues/organs/developmental stages. The independent gene expression datasets used for this study are provided in Supplementary Table S3. The expression values map to a color gradient from low (blue) to high (red) expression. Values are present using the log2 scale.

The close relationship between the *S. tuberosum* and Arabidopsis bZIP proteins within each group suggests that their biological roles may be shaped by their evolutionary history [11]. Therefore, we undertook a series of studies to determine whether the StbZIP gene expression profiles can be useful to predict their functions. First, we utilized large transcriptome datasets from publicly available repositories (detailed in Table S3) to produce hierarchical clustering of gene expression and to understand which *StbZIPs* express to higher levels in particular tissues (Figure 7). We obtained the gene expression profiles of the *StbZIP* genes in whole RH89-039-16 (RH) genotype plants and fourteen specific tissues: stem, leaf, petioles, roots, flowers, stamens, stolon, young and mature tubers, as well as tuber peel, pith, and cortex. *S. tuberosum* plants that were grown in the greenhouse, and tissues were harvested at the 12-leaf stage. Stamens were collected from open flowers, tubers were collected from plants after senescence, and sprouts were collected from harvested tubers. The data were compiled from five plants. The expression patterns differed among *StbZIP* gene families indicating members of multiple functional groups were mutually expressed in various tissues, as expected, given their role in a wide range of physiological processes. Notably, in many experimental studies, bZIP factors a one- or two-fold induction is significant for increased activity and, therefore greater levels of expression or repression point to important affiliations [42,43]. It is also notable that some gene pairs that arose by segmental duplication do not show the same expression patterns indicating functional divergence.

On a log2 scale of zero to 10, 34 bZIP genes show moderate expression levels (4.0–6.0) in one specific tissue but the trend toward lower expression (≤2.0) across most other tissues (Figure 7). Examples of moderate expression in one or more specific tissues include: (a) *StbZIP54, StbZIP56*, and *StbZIP62* in tuber sprouts; (b) *StbZIP37, StbZIP47*, and *StbZIP43* in roots and petioles, and (c) *StbZIP27* in roots and stolons. Interestingly *StbZIP64* is moderately expressed in the shoot apex, young tubers, and mature tubers. *StbZIP77* and *StbZIP66* highly (6.0–10.0) expressed in flowers and stamens and low (<2.0) in all other tissues (Figure 7). Group D contains many of the Arabidopsis TGA transcription factors (Table 1) which are essential for salicylic acid signaling, disease resistance, stress mitigation, and flower development. Many of the *S. tuberosum* orthologues in group D (*StbZIP47, StbZIP21, StbZIP65, StbZIP43, StbZIP46,* and *StbZIP74)* are moderately expressed in roots and vegetative tissues but show low expression in the majority of tissues. In group E the *AtbZIP34* is linked to pollen germination [4] and the data show that *StbZIP77* which is also in group E is highly expressed in the flower and stamen which might indicate a similar role (Figure 7).

Twenty-one factors were moderately expressed in the majority of tissues (>2.0 to 6.0). These included group A factors *StbZIP36, StbZIP40*, and *StbZIP66*; group C *StbZIP9;* group D *StbZIP20, StbZIP50,* and *StbZIP57*; in addition to the S1 group *StbZIP53,* and *StbZIP73.* Group I has eight StbZIP proteins, and four of these are moderate to highly expressed across a range of tissues: *StbZIP18, StbZIP30, StbZIP48,* and *StbZIP51*.

Seven bZIP factors were moderate or highly expressed (4.0–10.0) across a wide range of tissues and whole plants (Figure 7). By comparing the gene expression profiles with the comparative phylogeny of AtbZIP and StbZIP factors, we can make inferences about the functions of many StbZIP factors. Interestingly, group A *StbZIP3* which lacks a full bZIP domain is highly expressed across tissues. The S1 subgroup members *StbZIP4, StbZIP15, StbZIP44,* are highly expressed (6.0–10.0) in the shoot apex, flower, and tubers, whereas another S1 subgroup member, *StbZIP8* is only expressed in the flower and stamen. The *StbZIP60* and *StbZIP17* genes belonging to groups B and K are moderate to highly expressed in all tissues. Since these genes contribute to ER stress and protein quality control regulation it makes sense that they are expressed across tissues. Group D contains many of the Arabidopsis TGA transcription factors (Table 1), which are essential for salicylic acid signaling, disease resistance, stress mitigation, and flower development [36]. The group I *StbZIP49* is also in this category (Figure 7).

### 2.8. Expression of StbZIP Family Members Varies from Repression to Activation in Response to Five Hormones

**Figure 8:**
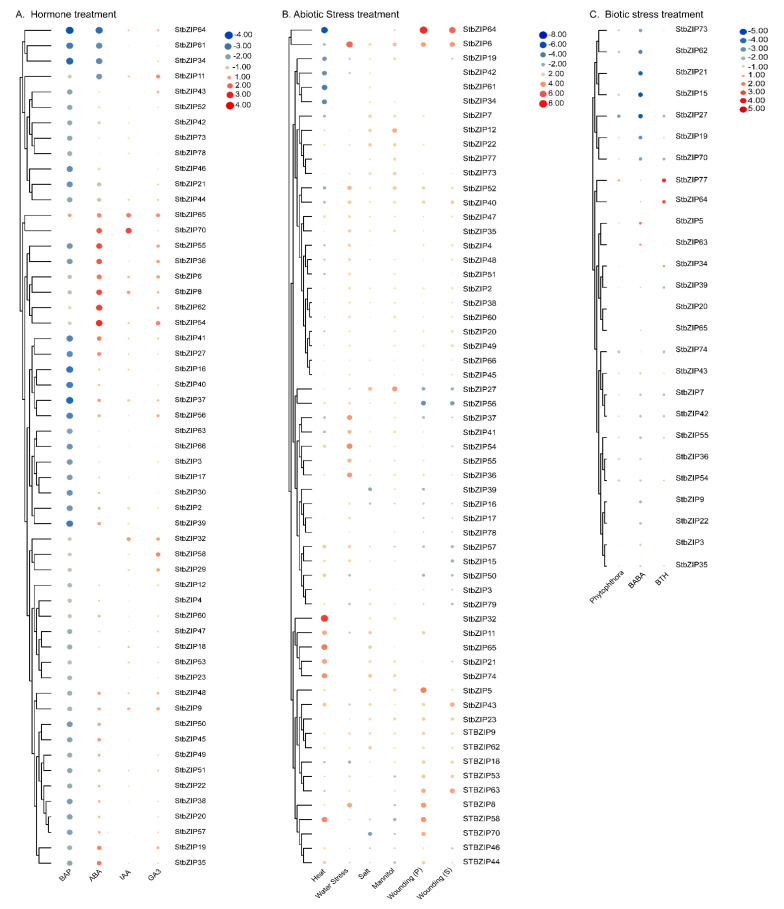
Heat map and hierarchical clustering of *StbZIP* transcript levels in response to the hormone, abiotic stress, and biotic stress. The independent gene expression datasets are detailed in Supplementary Table S3. The relative changes in bZIP gene expression levels were obtained following treatment with: (**A**) benzyl amino purine (BAP), abscisic acid (ABA), indole-3-acetic acid (IAA), or gibberellic acid (GA3); (**B**) heat, water stress, salt, mannitol, and wounding; (**C**) *P. infestans*, D,L-β-aminobutyric acid (BABA) or Benzo[1–3]thiadiazole-7-carbothionic acid (BTH). The log2 fold change values map to a color gradient from low (blue) to high (red) expression.

Seven important plant phytohormones regulate essential plant processes. To identify differentially expressed bZIP genes, we utilized a large transcriptome dataset obtained from a publicly available repository (detailed in Table S3). In vitro grown double monoploid potato plants were treated with 10 µM 6-benzyl amino purine (6-BAP), 50 µM ABA, 10 µM indole-3-acetic acid IAA or 50 µM gibberellic acid (GA3) (Figure 8A). The substance 6-BAP aids in the regulation of cell division and growth and is used in plant tissue culture media to support tissue regeneration. Following treatment with 10 µM 6-BAP, only one gene, *StbZIP65* showed a mild increase in transcript accumulation, while the remaining *StbZIP* genes were repressed. *StbZIP65* was also mildly activated by ABA, IAA, and GA3. Looking at the phylogeny *StbZIP65* is an orthologue of AtbZIP65/TGA10, a factor that contributes to plant growth and development, hydrogen peroxide-induced responses, and responses to bacterial infection [44,45].

As mentioned previously, ABA is also known as a “stress hormone” involved in adaptive responses to a wide range of abiotic and biotic stresses. Twelve factors were repressed in ABA treated leaves, eight that were unaffected, and the remaining factors were induced. Most notable, are four factors that are significantly repressed (−4.0 to −2.0) StbZIP64, StbZIP61, StbZIP34, and StbZIP11. The most highly induced genes (2.0 to 4.0) are also noteworthy, *StbZIP6, StbZIP8, StbZIP19, StbZIP35/AREB4, StbZIP36/AREB2, StbZIP54, StbZIP55, StbZIP62, StbZIP65, and StbZIP70*. Following IAA treatment *StbZIP8, StbZIP32, StbZIP65,* and *StbZIP70* were notably induced (1.0 to 4.0) while the majority of bZIP genes were mildly induced. After GA3 treatment there were eleven induced genes (1.0 to 4.0) *StbZIP6, StbZIP9, StbZIP11, StbZIP29, StbZIP32, StbZIP36/AREB2, StbZIP54, StbZIP55, StbZIP58,* and *StbZIP65* (Figure 8A).

### 2.9. Gene Expression Profiles Show Differential Responses to Various Abiotic and Biotic Stresses 

We investigated the *StbZIP* family genes that are responsive to environmental assaults using high-quality RNA-seq datasets from publicly available repositories (detailed in Table S3). We calculated the relative *StbZIP*s expression levels in tissues subjected to heat, water, salt, and osmotic (mannitol) stress. Many of the genes that are induced by abiotic stress are also involved in ABA responses, or oxidative stress and defense responses to pathogens. While we expected some similar patterns of gene induction, the transcriptome datasets also identified highly induced and highly repressed genes that were differentially affected by the various abiotic stresses (Figure 8B). For example, six genes were highly repressed following the heat treatment (≤−0.2): *StbZIP6, StbZIP19, StbZIP34, StbZIP42*, and *StbZIP61*. Genes that are highly induced by heat stress include *StbZIP32, StbZIP11, StbZIP18, StbZIP21, StbZIP43, StbZIP58, StbZIP65,* and *StbZIP74* (≥2.0), suggesting these are factors contribute to heat response signaling cascades in potato.

We examined the transcriptome profile derived from leaves following wilting (2 days without water) (Figure 8B). Seven factors were marginally repressed including *StbZIP11*, *StbZIP18*, *StbZIP19*, *StbZIP42*, *StbZIP43*, *StbZIP50*, and *StbZIP79*. At least eighteen factors were induced in response to water stress (≥2.0) including *StbZIP2, StbZIP4*, *StbZIP6, StbZIP8*, *StbZIP15*, *StbZIP35/AREB4*, *StbZIP36/AREB2*, *StbZIP38/AREB1*, *StbZIP40, StbZIP41*, *StbZIP47*, *StbZIP48*, *StbZIP52*, *StbZIP51*, *StbZIP54*, *StbZIP55*, *StbZIP57* and *StbZIP60*.

Salt and mannitol stress-induced similar genes to different levels (Figure 8B). For example, mannitol and salt stress led to high levels of *StbZIP12/ABL2* and *StbZIP27*. In addition, salt stress led to repression of *StbZIP*70 and mannitol repressed *StbZIP58*. The final treatment was wounding. The underside of a primary leaf was wounded, and then the same primary (P) leaf and an upper secondary (S) leaf were harvested after 24 hrs. Seventeen similar bZIP genes were induced in the P and S leaves. Unique to P leaves was the induction of *StbZIP7*, *StbZIP8*, *StbZIP11*, *StbZIP22*, *StbZIP36/AREB2*, *StbZIP44*, *StbZIP58*, and *StbZIP70*. There were two uniquely induced genes in S leaves, *StbZIP47* and *StbZIP48*. Eleven StbZIPs were repressed in both tissues.

For biotic stress, detached leaves were treated with *P. infestans* or D,L-β-aminobutyric acid (BABA) or benzo[1–3]thiadiazole-7-carbothionic acid (BTH) (Figure 8C). Following *P. infestans* treatment, only *StbZIP77* was induced while twelve factors were repressed: *StbZIP15, StbZIP19, StbZIP27, StbZIP36, StbZIP43, StbZIP42, StbZIP54, StbZIP55, StbZIP62, StbZIP70, StbZIP73*, and *StbZIP74*. Following treatment with BABA or BTH similar genes were repressed (Figure 8C). Only *StbZIP5* and *StbZIP63* were induced by BABA, while *StbZIP34, StbZIP39, StbZIP64*, and *StbZIP77* were induced by BTH.

To be comprehensive, we examined four additional datasets (Table S3) although these did not show significant changes in gene expression among *bZIP* family members. First, four cultivars of potato were subjected to 60 days of drought stress in the greenhouse and field plots, and there were no significant changes in *bZIP* gene expression (Table S4). In another dataset, plants were subjected to 0, 1, or 5 mg/kg cadmium, and then leaves or roots were harvested. There was no indication that cadmium treatment altered the expression levels of the *StbZIP* genes (Table S5). Goyer et al. (2015) inoculated Premier Russet (*Potato virus Y* strain O (PVY^O^)-resistant) and Russet Burbank (PVY susceptible) potatoes with PVY^O^ or PVY^NTN^ in a greenhouse and harvested leaves at 4 and 10 hrs (Table S6) [46]. Only *StbZIP34* was induced in both potatoes inoculated with PVY^O^ at 4 h. In another experiment, potato cyst nematode (*Globodera rostochiensis*) infective-juveniles were used to infect potato roots (Table S7), and there were no significant changes in bZIP gene expression. In the final dataset, potato tubers were treated with *P. infestans* (Table S8), and only *StbZIP5* and *StbZIP11* showed significant repression. Considering that these two genes were significantly induced in the leaves following abiotic stress or BABA treatment, it is interesting to see the opposite effect on *StbZIP5* expression in the tubers treated with *P*. *infestans*.

### 2.10. Gene Expression Profiles Show Differential Responses to Potato Virus X (PVX) Infection

Extensive investigations of ER stress responses to potexvirus infection in Arabidopsis determined that the viral encoded activates the unfolded protein response (UPR), leading to transiently increased accumulation of *AtbZIP60* and *AtbZIP17* transcripts between 2- and 5- days post-inoculation (dpi). Arabidopsis is a host for a related potexvirus*, Plantago asiatica mosaic virus*, and in *atbzip60, atbzip17*, and *atbzip60atbzip17* knock out mutant lines, virus accumulation is higher than in wild-type Arabidopsis plants [27]. In *N. benthamiana* plants that were silenced for *bZIP60*, the PVX and PVY infection levels were elevated compared to control leaves [47]. These combined data demonstrate a role for these two bZIP factors in suppressing virus accumulation in plants. In this study, we examined transcript profiles derived from leaves that were inoculated with PVX and then harvested at 2 and 3 dpi to identify the *StbZIP* factors whose expression is altered early in virus infection.

**Figure 9:**
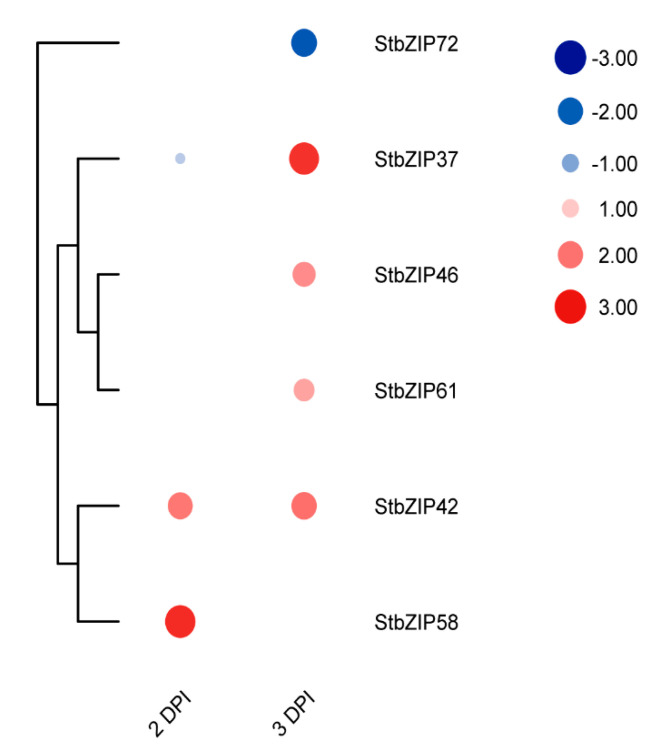
Heat map showing *StbZIP* transcript levels at 2 and 3 dpi with PVX. The transcriptomic analysis is BioPRoject PRJNA679820. The expression values (log2) map to a color gradient from low (blue) to high (red) expression. All values expressed in the heat map were significant (*p* <0.05).

RNA-seq analysis yielded ~453 million reads, and approximately 407 million reads were mapped to the reference potato genome with an average of 89.75% alignment rate (Table S9). We estimated the gene-level transcript abundance using RNA-Seq read counts. Among the differentially upregulated genes, six genes belonged to the potato bZIP family (Figure 9). We were surprised to see that *StbZIP72* was suppressed at 3 dpi, and five other *StbZIP* factors were elevated as an early response to infection, *StbZIP37/AREB3, StbZIP42, StbZIP46, StbZIP58,* and *StbZIP61*. These factors were not identified as suppressed or induced in the datasets involving biotic stressors BTH, BABA, or *P. infestans* suggesting these are virus-specific response factors. The *StbZIP72* belongs to group J, and the only Arabidopsis member belongs to this group, *AtbZIP62* was involved in oxidative stress and drought stress signaling [48]. The group A *StbZIP37* was assigned the synonym of *AREB3* in a recent study [22]. However, our analysis in this study identifies it as a putative ortholog of AtbZIP37/ABF3, a TF involved in the ABA-mediated signaling pathway that helps plants to acquire tolerance to drought, salt, cold head, and oxidative stress. The StbZIP37 likely has overlapping roles with other ABF/AREB family members [49,50].

## 3. Materials and Methods

### 3.1. Sequence Retrieval and Domain Characterization

We retrieved the *bZIPs* in the earlier assembly of double monoploid *S. tuberosum* group Phureja DM1-3 (genome assembly SolTub_3.0) and the Arabidopsis *bZIP*s (genome assembly TAIR10) from Ensemble Plants database release 44 (http://plants.ensembl.org/) based on the presence of a bZIP domain (InterPro ID #IPR004827) [20,21,51,52,53]. We also retrieved the *StbZIP*s in the latest genome assembly (ver 6.1) of potato (DM 1-3 516 R44) which we downloaded from the SpudDB (http://solanaceae.plantbiology.msu.edu/, last accessed 06.10.2020) [21]. We discovered *bZIPs* in the early assembly and recent assembly of potato and compared them. We determined that the new assembly has better coverage and quality and therefore gave the focus of our work to the latest assembly for further analysis of the *bZIP* family members.

Representative protein models were used to identify conserved protein domains obtained from the databases Pfam, ProSite Profiles, and SMART using InterPro v5.45–80.0 [54,55,56,57]. Only proteins with bZIP domains (InterPro ID #IPR004827) were further analyzed (Table S1). Candidate sequences were chosen based on an E-value of ≤1 × 10^−25^.

### 3.2. Phylogeny, Chromosomal Locations, Gene Duplications, and Intron/Exon Gene Structure Analysis

Multiple sequence analysis was carried out using MAFFT server v7 (https://mafft.cbrc.jp/alignment/server/, last accessed 07.10.2020) using the E-INS-i Iterative refinement method. TrimAl v1.2 was used to remove the unambiguous alignments using gappyout trimming mode. The Maximum likelihood (ML) phylogenetic tree was generated using IQ-TREE web server (http://iqtree.cibiv.univie.ac.at/; last accessed 10.10.2020) using JTT+G4 amino acid substitution model with 1000 ultrafast bootstraps and SH-aLRT branch test with 1000 replicates [58,59,60,61]. The ML phylogenetic tree was visualized using iTOL (v4) [62].

Potential gene duplication events were identified using MCScanX tools embedded in TBTools v1.051 [63,64]. This analysis used a tabulated BLASTP input file generated using legacy_blast.pl script of NCBI blast 2.90.+ using the following settings; blastp -e 1e-10 -b 5 -v 5 -m 8, and a gene finding format (GFF) file retrieved from the SpudDB (http://solanaceae.plantbiology.msu.edu/; last accessed 06.10.2020). The divergence analysis of paralogues *StbZIP* genes was carried out using the Ka/Ks calculator (Nei and Gojobori (NG) method) included in the TBTools ver 1.051.

The intron–exon structures and the intron phases of *bZIP* transcription factor genes were organized by aligning each CDS with their corresponding genomic sequences and visualized using Gene Structure Display Server (GSDS) 2.0 (http://gsds.gao-lab.org/, last accessed 08.10.2020) [65].

### 3.3. Amino acid Sequence Alignments and Analysis of the Conserved basic and Leucine-rich Domain

CLC Genomics Workbench 8.0 was used to visualize multiple protein sequence alignments. The basic region and C-terminal leucine-rich heptads were extracted into EXCEL spreadsheets (Supplementary Table S2) and conserved residues were manually analyzed according to [6,7]. The transmembrane domains were predicted using Protter v1.0, TMHMM Server v2.0 (http://www.cbs.dtu.dk/services/TMHMM/; last accessed 10.10.2020), and TOPCONS server [66,67]. The molecular weight (MW) of each StbZIP protein was calculated using COPid server (http://crdd.osdd.net/raghava/copid/; last accessed 10.10.2020) [68] and the isoelectric points were calculated using IPC calculator v1.0 using EMBOSS pKa set [69]. Conserved protein motifs among StbZIP families were analyzed using MEME Suite v5.2.0 (http://meme-suite.org/tools/meme; last accessed on 23.10.2020) [70]. The classic motif discovery mode was used to obtain the distribution pattern of zero to one occurrence per sequence (zoops). The maximum motif parameter was set to 50, and the motif width between 6 to 70 amino acids. Data were organized using Adobe Illustrator CC (2020).

### 3.4. Ab Initio Promoter Analysis

Promoter sequences representing 2000 bp from the transcription start site of *StbZIP* genes were extracted from the DM 1-3 516 R44-v6.1 assembly available at SpudDB (http://solanaceae.plantbiology.msu.edu/data/DM_1-3_516_R44_potato_genome_assembly.v6.1.fa.gz; last accessed on 26.10.2020). The locus annotations were provided (http://solanaceae.plantbiology.msu.edu/data/DM_1-3_516_R44_potato.v6.1.repr_hc_gene_models.gff3.gz; last accessed on 26.10.2020). CREs were analyzed against 417 CREs derived from monocotyledonous species (150), dicotyledonous species (263), and conifers (4) using the PlantCARE database (http://bioinformatics.psb.ugent.be/webtools/plantcare/html/) [71]. CREs were categorized based on their involvement in plant growth, hormonal regulation, and stress responses.

### 3.5. In Silico bZIP Gene Expression Analysis

Multiple independent gene expression datasets from the potato genotypes DM1-3 516 R44 genotype and RH89-039-16 reported in [20,72,73,74,75] and available in publicly accessible repositories were used in this study (Supplementary Table S3). Three datasets derived from RH genotype potato plants (Accession E-MTAB-552) were used to analyze *bZIP* expression profiles related to various tissues and developmental stages and were obtained from the Expression Atlas database (https://www.ebi.ac.uk/gxa/; last accessed 14.09.2020). The first dataset was obtained from RH plants that were grown in soil-filled pots in the greenhouse and at the 12th leaf stage, various tissues were sampled from five plants including fully expanded leaves, shoot apex, petioles, stem, mature tubers, and roots. Stamens were collected from fully open flowers. Non-tuberizing stolons were harvested and then young tubers that were ≤1 cm in size were collected for gene expression profiles at one week after the first swellings. Mature tubers were collected from senescing plants and then the peel, cortex, and pith were sampled. Tuber sprouts were obtained after storage in the dark for 3–4 months. The second dataset was obtained from RH plants that were grown in vitro on media. The third dataset was from water-stressed RH plants that were grown in the greenhouse and denied water for two days. Then the 2nd–4th fully expanded, wilted leaves were harvested. The *bZIP* transcriptomic profiles of DM potato (Accession E-MTAB-552, −553, −554, −555) following treatment with various hormones or abiotic stressors (Supplementary Table S3) were downloaded from SpudDB (http://solanaceae.plantbiology.msu.edu/index.shtml; DM_RH_RNA-Seq_FPKM_expression_matrix_for_DM_v4.03_132dec2013_desc.xlsx; last accessed date 14.09.2020) [20,73]. These gene expression datasets were generated using in vitro grown DM plants maintained at 22 °C day/18 °C night with a 16 h photoperiod. Plants were treated for 24 h and then roots and shoots were harvested together for RNA extraction. Transcriptome datasets were obtained following treatment with abscisic acid (ABA; 50 µM), indole-3-acetic acid (IAA; 10 µM), gibberellic acid (GA3; 50 µM), and 6-benzyl amino purine (6-BAP; 10 µM). Other abiotic stress conditions included heat (35 °C), salt (150 mM NaCl), and mannitol (260 µM) [20]. For wounding, the bottom two leaflets were mechanically injured, and then the primary leaflets and secondary non-wounded leaflets were harvested at 24 h.

The bZIP transcriptomic profiles of DM potato under biotic stress (Supplementary Table S3**)** were also retrieved from SpudDB (E-MTAB-552, EMTAB-4301, and E-MTAB-5215) [20,73,74]. In these experiments, six leaves were detached from greenhouse-grown DM plants and then spray-inoculated with *P. infestans* (Pi isolate US8: Pi02-007) using 0.5 mL of inoculum concentration of 30,000 sporangia/mL, acibenzolar-S-methyl (BTH; 100 mg/mL) or DL-B-amino-n-butyric acid (BABA; 2 mg/mL), and a mock inoculation was also conducted. Inoculated leaves were kept in the dark at room temperature for 8–10 h and then under lights for seven days. Infection experiments were repeated three times. Tissues from inoculated and mock-inoculated leaves were collected at 24, 48, and 72 hrs. for RNA isolation. RNAs were pooled.

One independent dataset (E-GEOD-77826) belongs to a study of greenhouse and field-grown plants belonging to four commercial cultivars ‘Alegria’, ‘Milva’, ‘Desiree’, and ‘Saturna’ [75]. Plants were subjected to drought stress at 12 days after transfer to pots in the greenhouse by withholding water. After 20 days plants received 30% of the water given to control plants. RNA was extracted from leaves of two to four replicates per cultivar. Four experimental datasets (E-MTAB-771, E-MTAB-4301, E-MTAB-5215, SRP058212, and SRP058230) were analyzed to obtain gene expression patterns following treatment with cadmium, *P. infestans*, *G. rostochiensis*, and potato virus Y (Supplementary Table S2).

Expression analysis was performed using each independent dataset. For hierarchical clustering, we relied on TBTools ver 1.0532 to perform complete linkage clustering and Euclidean distance measures. Then the data were visualized using the HeatMap tool built into TBTools ver 1.0532 [63].

### 3.6. PVX Inoculation of Potato Leaves for Transcriptomic Analysis

*S. tuberosum* cultivar “Russet Norkota” was multiplied by cuttings or in vitro on Murashige and Skoog medium. Rooted cuttings were grown in a growth room with a 12 h photoperiod at 20 °C for four weeks. The PVX-GFP infectious clone is maintained in *Agrobacterium tumefaciens* strain GV3101 [76]. A fresh culture originated from a single colony was used for to agro-infiltrate plants using standard methods [27]. Inoculated *Nicotiana benthamiana* plants grown under 12 h light at 20 °C. Then upper leaves were harvested after the appearance of symptoms (2 weeks), ground 1:10 (*w*/*v*) in 0.01 M phosphate buffer (pH 7.0), centrifuged at 6000 rpm for 5 min, and then the soluble phase (sap extract) was stored at −80 °C. Standard infectivity assays were carried out to estimate the amount of infectious virus in the sap preparation used, to ensure future reproducibility [77]. *Chenopodium quinoa* leaves (n = 6) were rub-inoculated with 20 µL of sap and the numbers of chlorotic foci were counted after 7–12 days. The average number of foci across three leaves was 44. After determination of the infectivity, three potato leaves (cultivar ‘Russett Norkota’) were mechanically inoculated with 20 µL of sap after dusting with carborundum. Mock treatment was carried out using only the phosphate buffer (three biological replicates for PVX infected and mock treated plants). To study the transcriptomic changes in early infected leaves at 2 and 3 dpi, inoculated leaf samples were collected and immediately frozen in liquid nitrogen, and stored at −80 °C freezer for use in transcriptomic studies.

### 3.7. Transcriptomic Analysis

To investigate early changes in gene expression, we used the inoculated russet potato leaves that were harvested at 2 dpi and 3 dpi following PVX-GFP inoculation and mock treatment, and were stored at −80 °C. Three frozen leaves from each PVX-GFP inoculated or mock treated russet potato plants were combined and homogenized for RNA extraction (3 biological replicates). The RNeasy Mini Kit (Qiagen Co., Hilden, Germany) was used to extract total RNA. RNA purity was assessed using Epoch 2 Microplate Spectrophotometer (BioTek Instruments Inc., VT, USA). All samples produced A_260_/A_280_ ratios ranging between 1.9–2.1. RNA integrity was assessed using Agilent 2100 bioanalyzer (Agilent Technologies, Palo Alto, CA, USA) and all samples had an RNA integrity number (RIN number) >7.3.

The mRNA purification, fragmentation, cDNA synthesis, second-strand synthesis, adapter ligation, cDNA library purification, and transcriptomic sequencing were performed at the Beijing Genomics Institute (BGI, Shenzhen, China) using the BGISEQ-500 platform. BGI performed PE150 strand-specific library preparation, generated raw data, and provided clean reads as follows. First, the poly-A-containing mRNA was purified using oligo(dT)-coupled magnetic beads. Then mRNA fragmentation was carried out using divalent cations under elevated temperature. The cleaved fragments were converted into the first-strand cDNA using reverse transcriptase and random primers. Then second-strand cDNA synthesis was used by applying DNA polymerase I and incorporating dUTP (2′-deoxyuridine 5′-triphosphate) in place of dTTP (2′-deoxyguanosine 5′-triphosphate) to generate double stranded cDNA. The final cDNA library was generated by purifying and PCR enriching the product from the earlier step. Using a rolling-circle replication mechanism, single-stranded DNA circles containing DNA nanoballs were generated. Then the DNA nanoballs were then loaded into patterned nanoarrays, and paired-end reads of 150 bp were generated with the BGISEQ-500. The raw data with adapter sequences or low-quality sequences were filtered using SOAPnuke (v2.1.0) [78]. FASTQC was used to assess read qualities (version 0.11.9). The subsequent analysis returned clean reads.

Reference guided mapping was carried out using the latest genome assembly (DM v6.1) of potato [21]. Reads from PVX-infected and mock-treated datasets at 2 and 3 dpi were aligned to the potato reference genome (DM v6.1) using HISAT2 (v2.2.0). The SAM files were converted to BAM files and indexed using SAMtools (v1.9) [79]. Transcripts assembly and abundance were determined using StringTie (v2.1.4) [80] and using the annotations obtained from the reference genome (DM v6.1). Then the results were converted to DESEQ2 format using prepDE.py python scripts available with the program. Differential sequence analysis was carried out using DESEQ2 (v1.28.1) in RStudio (v1.3.959) [81]. Differentially regulated *bZIP* genes with ≤−1.2 or ≥1.2 log^2^-fold difference with an adjusted *p*-value of ≤0.05 at each 2 dpi and 3 dpi were used for the visualization using the HeatMap tool built into TBTools v1.0532 [63].

## 4. Conclusions

In this study, we identified 80 *bZIP* genes in *S. tuberosum*. The results revealed the structural and functional diversification of *bZIP*s in potato. It is evident that gene duplications have contributed towards the expansion of the bZIP family. During its evolutionary trajectory, the *bZIP* gene family expanded to many groups and these groups were mostly conserved between the potato and Arabidopsis, indicating the functional importance of the family in growth, development, and stress responses. We also identified a novel bZIP gene group (N) consisting of four genes, while group M of Arabidopsis has been lost in potato. The presence of multiple members in each group (except for group K) indicates the functional redundancy and differential expression patterns observed in our study. The identification of the *bZIP* gene family in potato will act as the first step towards structural and functional characterization of the bZIP family. Together with the recent reports describing quantitative trait loci associated with the key developmental and stress-related traits [82,83,84,85,86], these findings will help breeders to develop future-proof potato varieties with enhanced yield potential.

## Figures and Tables

**Table 1:** AtbZIP and StbZIP family members organized by functional.

Group	Gene Name	Locus ID	Synonym	GenBank	Group	Gene Name	Locus ID	Synonym	GenBank
A	AtbZIP12	AT2G41070	DPBF4	AF334209	G	AtbZIP16	AT2G35530		NM179917
	AtbZIP13	AT5G44080				AtbZIP41	AT4G36730	GBF1	NM179179.3
	AtbZIP14	AT4G35900	FD	NM119756.5		AtbZIP54	AT4G01120	GBF2	NM116342.3
ABA Responsive	AtbZIP15	AT5G42910	MBD2	NM123656.2		AtbZIP55	AT2G46270	GBF3	NM001337182.1
Flowering time	AtbZIP27	AT2G17770	FD paralog	NM127331.3		AtbZIP68	AT1G32150		NM102948.4
Seed germination	AtbZIP35	AT1G49720	ABF1	NM001198254.2		StbZIP16	Soltu.DM.08G003670		
Abiotic Stress	AtbZIP36	AT1G45249	ABF2, ATAREB1	NM001333256.1		StbZIP24	Soltu.DM.02G006750		
	AtbZIP37	AT4G34000	ABF3	NM001342246.1		StbZIP41	Soltu.DM.02G025470		
	AtbZIP38	AT3G19290	ABF4, AREB2	NM001203005.2		StbZIP54	Soltu.DM.05G019830		
	AtbZIP39	AT2G36270	ABI5	NM129185.4		StbZIP55	Soltu.DM.01G034570		
	AtbZIP40	AT1G03970	GBF4	NM100278.3		StbZIP68	Soltu.DM.08G021830		
	AtbZIP66	AT3G56850	AREB3	NM115544.3	H	AtbZIP56	AT5G11260	HY5	NM001343175.1
	AtbZIP67	AT3G44460	DPBF2	NM114314.5	Anthocyanin Accum.	AtbZIP64	AT3G17609	HYH	NM001084700.2
	StbZIP2	Soltu.DM.01G005870			Light Responsive,	StbZIP56	Soltu.DM.08G011730		
	StbZIP3	Soltu.DM.01G006940			I	AtbZIP18	AT2G40620		NM129624.5
	StbZIP12	Soltu.DM.10G030340	ABL2			AtbZIP29	AT4G38900		NM001036733.4
	StbZIP13	Soltu.DM.04G027170				AtbZIP30	AT2G21230		NM001335735.1
	StbZIP14	Soltu.DM.02G023460				AtbZIP31	AT2G13150		NM126912.1
	StbZIP27	Soltu.DM.02G005680				AtbZIP32	AT2G12940	UNE4	NM126904.6
	StbZIP35	Soltu.DM.11G016910	AREB4			AtbZIP33	AT2G12900		NM126901.1
	StbZIP36	Soltu.DM.04G033590	AREB2			AtbZIP51	AT1G43700		NM103495.4
	StbZIP37	Soltu.DM.10G015000	AREB3			AtbZIP52	AT1G06850		NM001331671.1
	StbZIP38	Soltu.DM.01G047570	AREB1			AtbZIP59	AT2G31370		NM001336329.1
	StbZIP39	Soltu.DM.09G003620	ABI5			AtbZIP69	AT1G06070		NM100488.4
	StbZIP40	Soltu.DM.01G043800				AtbZIP71	AT2G24340		NM127996.1
	StbZIP66	Soltu.DM.10G025990	ABL1			AtbZIP74	AT2G21235		NM179679.2
B	AtbZIP17	AT2G40950		NM129659.3		StbZIP18	Soltu.DM.06G011870		
ER Stress	AtbZIP28	AT3G10800		NM111917.5		StbZIP29	Soltu.DM.01G050330		
Responsive	AtbZIP49	AT3G56660		NM115525.2		StbZIP30	Soltu.DM.04G036300		
	StbZIP17	Soltu.DM.10G029910				StbZIP48	Soltu.DM.04G026570		
	StbZIP28	Soltu.DM.04G021020				StbZIP49	Soltu.DM.06G017610		
	StbZIP33	Soltu.DM.04G020920				StbZIP51	Soltu.DM.04G026750		
	StbZIP67	Soltu.DM.08G019490				StbZIP52	Soltu.DM.06G011430		
	StbZIP70	Soltu.DM.08G019530				StbZIP69	Soltu.DM.06G014230		
	StbZIP71	Soltu.DM.08G019590			J	AtbZIP62	AT1G19490		NM101806.3
C	AtbZIP9	AT5G24800		NM122389.4		StbZIP62	Soltu.DM.10G024960		
C/S1 Regulatory Network, Energy Starvation, Seed Development	AtbZIP10	AT4G02640		NM001340389.1		StbZIP72	Soltu.DM.09G003280		
	AtbZIP25	AT3G54620		NM115319.4	K	AtbZIP60	AT1G42990		NM103458.3
	AtbZIP63	AT5G28770		NM122760.4	ER stress Responsive	StbZIP60	Soltu.DM.04G038150		
	StbZIP9	Soltu.DM.08G002700			M	AtbZIP72	AT5G07160		NM120798.2
	StbZIP10	Soltu.DM.08G008380			N	StbZIP25	Soltu.DM.03G005150		
	StbZIP63	Soltu.DM.01G036510				StbZIP26	Soltu.DM.03G005200		
D	AtbZIP20	AT5G06950	AHBP-1B; TGA2	NM001036768.2		StbZIP59	Soltu.DM.06G032460		
SA Responsive	AtbZIP21	AT1G08320	TGA9	NM001331769.1		StbZIP75	Soltu.DM.11G017940		
or	AtbZIP22	AT1G22070	TGA3	NM102057.4	S	AtbZIP1	AT5G49450		NM124322.3
Early Flowering	AtbZIP26	AT5G06960	OBF5; TGA5	NM203016.2	Includes the C/S1 Regulatory Network	AtbZIP2	AT2G18160		NM127373.2
	AtbZIP45	AT3G12250	TGA6	NM202564.2		AtbZIP3	AT5G15830		NM121588.3
	AtbZIP46	AT1G68640	PAN	NM105536		AtbZIP4	AT1G59530		NM104646
	AtbZIP47	AT5G65210	TGA1	NM125919.3		AtbZIP5	AT3G49760		NM114836.3
	AtbZIP50	AT1G77920	TGA7	NM106441.4		AtbZIP6	AT2G22850		NM127850.3
	AtbZIP57	AT5G10030	TGA4	NM001343084.1		AtbZIP7	AT4G37730		NM119935.3
	AtbZIP65	AT5G06839	TGA10	NM001203315.2		AtbZIP8	AT1G68880		NM105562.3
	StbZIP1	Soltu.DM.01G005540				AtbZIP11	AT4G34590		NM119625.3
	StbZIP20	Soltu.DM.11G019010				AtbZIP42	AT3G30530		NM113954.2
	StbZIP21	Soltu.DM.06G029750				AtbZIP43	AT5G38800		NM123241.3
	StbZIP43	Soltu.DM.04G022450				AtbZIP44	AT1G75390		NM001084357.2
	StbZIP45	Soltu.DM.10G026630				AtbZIP48	AT2G04038		NM126441.1
	StbZIP46	Soltu.DM.05G002740				AtbZIP53	AT3G62420		NM116107.2
	StbZIP47	Soltu.DM.04G007700				AtbZIP58	AT1G13600		NM101230.4
	StbZIP50	Soltu.DM.12G007270				AtbZIP70	AT5G60830		NM125476.1
	StbZIP57	Soltu.DM.04G028540				AtbZIP75	AT5G08141		NM125476.1
	StbZIP65	Soltu.DM.10G029320				StbZIP4	Soltu.DM.01G024850		
	StbZIP74	Soltu.DM.10G027000				StbZIP5	Soltu.DM.03G005290		
	StbZIP80	Soltu.DM.11G021800				StbZIP6	Soltu.DM.02G027390		
E	AtbZIP34	AT2G42380		NM001336969.1		StbZIP7	Soltu.DM.04G032270		
	AtbZIP61	AT3G58120		NM115674.4		StbZIP8	Soltu.DM.01G040220		
	AtbZIP76	AT1G58110		NM001036128.2		StbZIP11	Soltu.DM.02G024680		
	AtbZIP77	AT1G35490		NM001333158.1		StbZIP15	Soltu.DM.01G049720		
	AtbZIP78	AT4G06598		NM001340558.1		StbZIP22	Soltu.DM.02G006290		
	AtbZIP79	AT5G04840		NM120566.6		StbZIP31	Soltu.DM.03G006600		
	StbZIP34	Soltu.DM.12G028390				StbZIP32	Soltu.DM.04G000200		
	StbZIP61	Soltu.DM.07G020410		XP_006348282		StbZIP42	Soltu.DM.02G030370		
	StbZIP64	Soltu.DM.07G023890				StbZIP44	Soltu.DM.04G035840		
	StbZIP76	Soltu.DM.10G011180				StbZIP53	Soltu.DM.06G000140		
	StbZIP77	Soltu.DM.11G012470				StbZIP58	Soltu.DM.05G001710		
	StbZIP78	Soltu.DM.01G042790				StbZIP73	Soltu.DM.10G016240		
	StbZIP79	Soltu.DM.11G007430							
F	AtbZIP19	AT4G35040		NM001342322.1					
	AtbZIP23	AT2G16770		NM001335489.1					
	AtbZIP24	AT3G51960		NM001339543.1					
	StbZIP19	Soltu.DM.10G017560							
	StbZIP23	Soltu.DM.01G051130							
Functional Groups, Gene ID, Locus ID, Synonyms and NCBI accessions are provided for all genes used in this study. The color coding for the functional groups correspond to the colors in the legends in Figure 1.									

**Table 2:** The nonsynonymous (Ka) and synonymous (Ks) substitution ratio (Ka/Ks) test for 22 gene pairs.

Duplicated Gene Pair	Ka	Ks	Ka/Ks
StbZIP55/StbZIP54	0.18	0.63	0.29
StbZIP38/StbZIP37	0.19	0.67	0.29
StbZIP38/StbZIP36	0.39	2.86	0.14
StbZIP29/StbZIP30	0.36	1.88	0.19
StbZIP23/StbZIP19	0.17	0.67	0.26
StbZIP27/StbZIP14	0.4	1.33	0.30
StbZIP14/StbZIP37	0.69	1.43	0.48
StbZIP11/StbZIP44	0.26	1.17	0.23
StbZIP6/StbZIP7	0.44	1.39	0.32
StbZIP6/StbZIP5	0.26	0.87	0.30
StbZIP5/StbZIP6	0.26	0.87	0.30
StbZIP7/StbZIP6	0.44	1.39	0.32
StbZIP36/StbZIP38	0.39	2.86	0.14
StbZIP44/StbZIP11	0.26	1.17	0.23
StbZIP30/StbZIP29	0.36	1.88	0.19
StbZIP54/StbZIP55	0.18	0.63	0.29
StbZIP61/StbZIP34	0.25	1.1	0.23
StbZIP37/StbZIP38	0.19	0.67	0.29
StbZIP19/StbZIP23	0.17	0.67	0.26
StbZIP66/StbZIP12	0.15	0.57	0.26
StbZIP12/StbZIP66	0.15	0.57	0.26
StbZIP34/StbZIP61	0.25	1.1	0.23

## Data Availability

Transcriptomic data are available on the National Center for Biotechnology Information Sequence Read Archive (NCBI SRA: https://www.ncbi.nlm.nih.gov/sra) under the bioproject PRJNA679820.

## Supplementary Materials

The following are available online at https://www.mdpi.com/1422-0067/22/1/253/s1; Figure S1. Motif sequences identified by MEME Tools; Table S1. Compilation of *StbZIP* genes, their identifiers, and protein properties; Table S2. Brief alignment of the basic region and leucine-rich domain of Arabidopsis and *S. tuberosum* bZIP factors; Table S3. Compiled independent transcriptome datasets used for in silico bZIP gene expression analysis; Table S4. Relative expression levels of greenhouse (GH)and field-grown (FG) plants treated with 60 days of drought stress; Table S5. Relative gene expression profile of potato plants treated with 0, 1, and 5 mg/kg cadmium; Table S6. Relative gene expression profiles of early stages of potato virus Y infection in resistant and susceptible potato varieties; Table S7. Relative gene expression profile of potato plants treated with *G. rostochiensis*; Table S8. Relative gene expression profiles of *P. infestans* inoculated potatoes and RB transgenic potatoes; Table S9. Summary of the RNA-sequencing read data.
