# Supplementary figures and images for: Insight into the bZIP Gene Family in Solanum tuberosum: Genome and Transcriptome Analysis to Understand the Roles of Gene Diversification in Spatiotemporal Gene Expression and Function
Source: Int J Mol Sci. 2020 Dec 29;22(1):253. doi: 10.3390/ijms22010253 (PMC7796262; doi:10.3390/ijms22010253)

## N-terminal Motifs

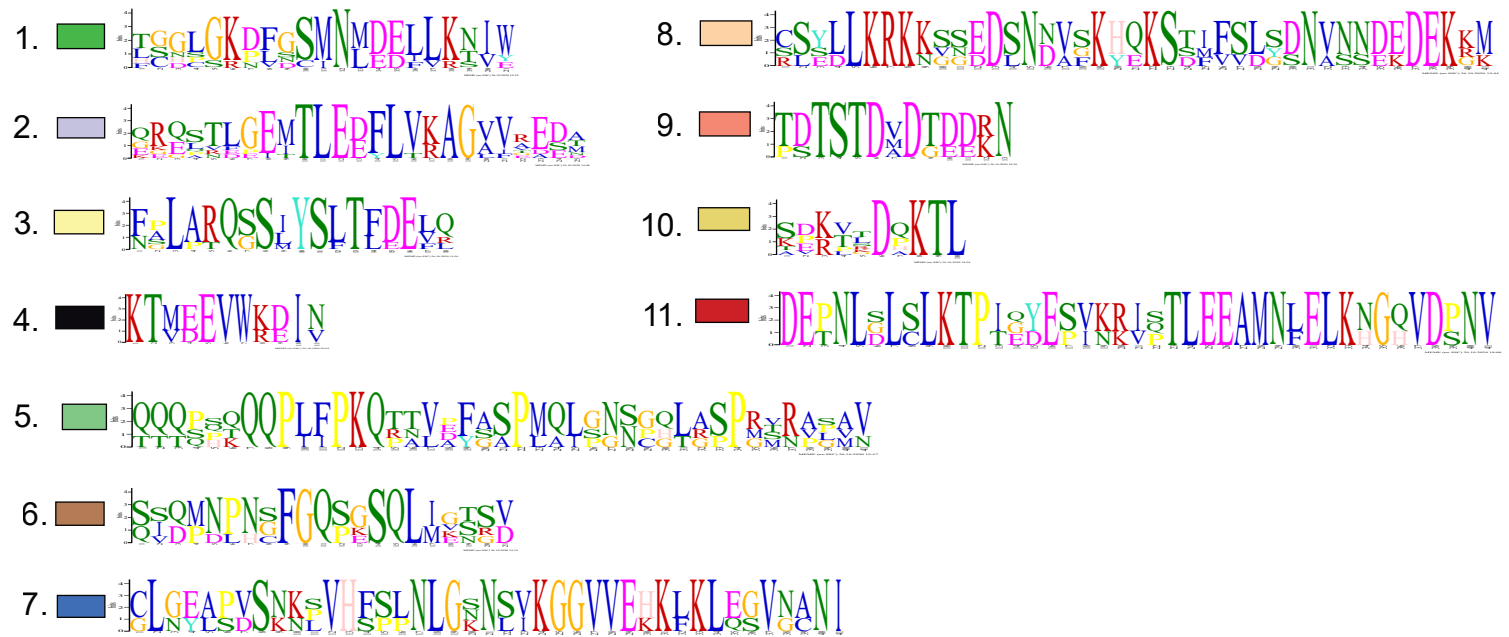

## Basic and Leucine Rich Motifs

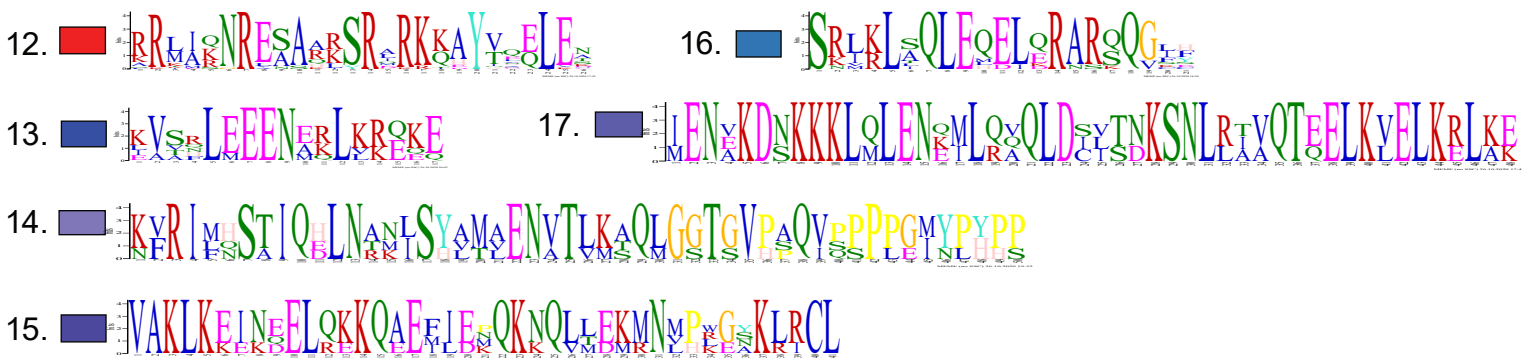

## C-terminal motifs

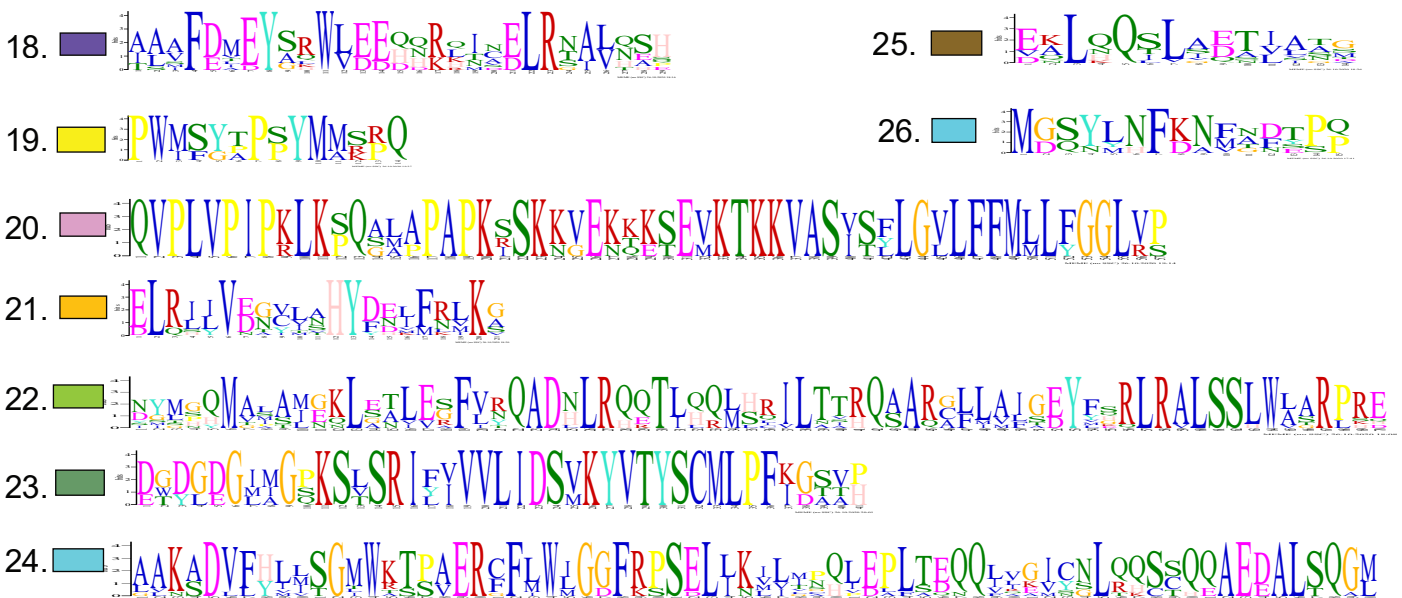

Supplement: Supplementary file 1 [file ijms-22-00253-s001.zip › Supplementary Figure 1.pdf]
